# Controlled to uncontrolled drug use: The impact of Covid-19 among young people in the UK

**DOI:** 10.1080/00187259.2024.2379308

**Published:** 2024-08-15

**Authors:** Hayley Murray

**Affiliations:** Knowledge, Technology and Innovation Group, Social Science Group, Wageningen University and Research, Wageningen, the Netherlands

**Keywords:** Covid-19, harm reduction, young people, cannabis, alcohol

## Abstract

The Covid-19 pandemic lockdown had a profound impact on British young adults’ drug using lives. Overnight, participants found themselves unable to access the protective mechanisms, specifically peer groups and routines on which they had come to rely to control and maintain pleasure with their drug use. The resulting analysis from online semi-structured qualitative interviews with 14 young people exposes a trend in drug use patterns. Substances that are easily intertwined in their daily lives and the conditions of lockdown, such as cannabis, alcohol, and cocaine were used more frequently and more habitually. Despite a perception of low risk due to prevalent use, these substances pose a heightened risk of dependency. In this article, I argue that because they were socially isolated and without protective mechanisms, such as peer support or daily routines, participants incorporated these drugs into their work-from-home regimes. This gave rise to a potential lack of control of their use. This insight contributes to enhancing a nuanced understanding of (un)controlled drug use among young people and the factors that influence this.

## IMPLICATIONS

This research underscores the need for enhanced support systems for recreational drug users. Key areas include establishing peer support networks, ensuring accessible mental health services (both online and in-person), and providing harm reduction tips for high dependency drug use through public health campaigns. External factors like routines and peer support are vital in managing drug use, and their absence during lockdowns created dangerous conditions, especially for young people with mental health struggles. Promoting sustainable routines and comprehensive mental health support is essential to address uncontrolled drug use and the impacts of social isolation and loneliness.

## Social Media Statement

The Covid-19 pandemic stripped young British recreational drug users of protective mechanisms, such as peer groups and routines, leaving regular users isolated and vulnerable to habitual and uncontrolled cannabis and alcohol use.

The relationship between social isolation and drug use is closely linked through a combination of psychological, social, and behavioral factors. For those who face isolation, elements such as coping mechanisms, lack of social support and accountability, changes in routine, and strains on mental health, can lead to increased drug use. Researchers and policy makers have consistently shown ongoing concern about how these two elements impact each other (Copeland et al. 2018; Nawi et al. 2021). The Covid-19 pandemic only amplified these concerns. During the United Kingdom’s three national lockdowns,^1^ more than at any other point in the pandemic, young people faced abrupt changes to their social lives and daily routines. The restrictions meant that young people went from seeing colleagues at work and friends at school and going on dates, to solely interacting with the people (if any) with whom they were sheltering-in-place. Additionally, during these stretches of time, young people experienced an upheaval of their daily routine and went from an active pace of commuting to work, having appointments and social engagements, to being forced to conduct their daily routine in one location.

Young people tend to seek out routinized practices which create “tacit knowledge about how to navigate everyday life; normative signposts for what to do and not do” (Bengtsson and Ravn 2021: 1). What would it mean for those who no longer had a routine to hold onto, for a sense of safety? These changes are important to consider because we know that peers and daily routine have a strong influence on young people’ drug use (Hardon 2021; Beard and Wolff 2022; Coggans and McKellar 2009). What would happen if these key factors of socialization and rhythms were threatened? Drug use is largely influenced by social controls like rituals and rules, reflecting group norms and peer networks (Zinberg 1986). What would that mean for those who spent lockdown with someone who used drugs more frequently than those who spent it alone?

Young people often articulate that because they use drugs socially, not alone, their use can be seen as recreational and not in the direction of dependency (Nichter and Taylor 2021). However, during lockdown, many young people were now getting high alone. How did that impact their understanding of their drug use? Or, as the distinction between work and play became vague, how did this upset the regular rhythm of their drug use? Did young people broaden their understanding of the acceptability of their drug use? Or suddenly faced with a sharp lack of opportunities to socialize, did young people turn to drug use to ‘shake up’ their monotonous days?

While there is ample literature on the socialness of drug use (Hunt and Evans 2008; Duff 2012; Mayock, Corr, and O’Sullivan 2017), the research on which this article is focused, and on which it builds, is what happens when young people use drugs in precarious situations. The Covid-19 pandemic created an uncertain setting for young people, who faced challenges adapting to life under lockdowns, restrictions, and working from home. Studies and policies alike both point to the pandemic’s detrimental effects of the isolating conditions of lockdown on young people’s mental health (Harbers, Hulshof, and Schaink 2020; YoungMinds 2021; Townsend 2020; Brenan 2020; Twenge and Joiner 2020). It also stripped young people who use drugs of their protective mechanisms, including peer networks, and produced a setting where some young people became more vulnerable to increased drug use (Layman et al. 2022; Rogés et al. 2021, Dumas, Ellis, and Litt 2020). Current research on young people’s drug use practices during the Covid-19 pandemic suggests that they may be at a higher risk of dependency, uncontrolled use, and other drug-related harms, some of which are yet to be fully understood (Turner et al. 2021; Guest, Coope, and Oram 2021; Benschop, van Bakkum, and Noijen 2021; Woo, Bhalerao, and Bhatia 2020; Hawke et al. 2020; Trimbos Instituut 2020). These risks also appear when considering drug use practices while being confined to the home. Participants turned towards substances that are well-suited for solitary enjoyment, not attached to a particular activity, and have a higher rate of dependency (such as cannabis or alcohol) and away from those that are well-suited for social environments, associated with activities such as raves, and are generally used less habitually (such as MDMA or amphetamines), echoing a claim which has been made elsewhere (Dumas, Ellis, and Litt 2020). These studies provide us with nuanced knowledge about the impact of public health crises and the resulting uncertainty and isolation on young peoples’ drug use and mental health. It also provides insight into the factors that contribute to resiliency in young people during difficult and transitional periods. Taking these elements into consideration, I explore how young people, isolated from their peers and working from home, were coping with the changes brought about by the pandemic.

In this article, I draw on longitudinal qualitative interviews with young people who use drugs in the Greater London area and who were aged 22 to 25 years at the onset of the study. The initial study (2018–2019) tracked participants’ drug using lives over 18 months with thematic interviews held every six months to better understand why some individuals develop ‘problematic’ drug use and others do not. In the fall of 2020, there was an opportunity to conduct additional interviews and explore participants’ drug use into the pandemic. This provided me with a vantage point to extend our understanding about which factors lend themselves to (un)controlled drug use, which, ultimately, can be applied to better support young people who use drugs to do so more safely. My goal with this study was to find out how my interlocutors, now aged between 24 and 27, were coping with the conditions of the pandemic. The motivation for continued research sprung from initial findings from the longitudinal study which indicated that participants sought out and relied on a multitude of structures, values, and daily routines to regulate and control their drug use (Murray 2023). An extension of this finding was the (largely unattainable) balancing act that participants continuously engage with in the face of challenges such as one’s life stages, drug choices, and context.

In the initial study, we learned that young people create protective mechanisms to help control and self-regulate their drug use. The popularly held damaging belief found in literature is that drug use by youth is generally risky, problematic, or mismanaged (Banken 2004; Kokkevi et al. 2007; Salvatore 2018). In contrast, this finding revealed that young people have a plethora of ways to control their drug use by focusing on maximizing the pleasure and minimizing the risks, a sentiment that has been pointed out in recent ethnographies on young drug takers (Hardon and Hymans 2016; Van Schipstal et al. 2016; Hardon 2021; Hardon et al. 2020). Specifically, they created and relied on certain safeguards, such as commitments, obligations, goals, expectancies, and responsibilities, embedded in their daily routines to help control drug use (Decorte, 2001). These safeguards were all easily accessible to participants pre-pandemic. For example, Klaas (25), a regular cannabis user, made rules about when it was unacceptable to be high, such as before work or seeing his girlfriend’s family. This regular routine helped him ensure that his drug use would take place only at certain times of the day or week, giving him a sense of control over his use. If Klaas relied on protective mechanisms to control his drug use and these were no longer available due to the conditions of the pandemic, and finding a balance already proved challenging, then how did he, and others like him, manage their drug using lives, if at all?

Understanding the drug using lives of young people can be further enhanced by utilizing the findings from the collective of ethnographers from the ChemicalYouth project (Hardon 2021). Through our multi-sited ethnographies, we found that young people, together with the caring nature of their network of peers, tinker with their drug use practices as a form of controlled self-regulation. This, we have argued, is a form of ‘harm reduction from below’, which builds on young peoples’ pre-existing interest in mitigating risks related to drug use and prioritizes pleasure over harm (Van Schipstal et al. 2016; Mandler 2016; Hardon 2021). Transferring this knowledge to the current study improves our understanding of the multitude of protective mechanisms normally available to young people. Highlighting the importance of social connectivity, we argue that harm reduction from below “can best be seen in the practices of sharing around drug use and in the caring for the larger community of drug-using peers” (Van Schipstal et al. 2016, 199). Safer drug use may be based on what people are seeing around them: Is it normal to use in excess or is that frowned upon? In this way, others’ practices are reflected to an individual, who can then determine what their boundaries are and when they are crossed. The drug use practices and patterns that one would see at parties or among a group of friends is important, as these (unconsciously) shape our understanding of what is considered acceptable, controlled, and enjoyable, and what is not. Accordingly, if we consider the social and physical isolation of participants, there is little availability for ‘sharing’ and ‘caring’ in their drug use practices during the pandemic.

At the same time, self-regulation techniques and structures are also seen to be a foundation of ‘harm reduction from below’, where young people created their own ways of controlling and monitoring their drug use, independent from their peers. This was observed in the creation of goals, responsibilities, and activities (Van Schipstal et al. 2016, 211). But as noted above, the pandemic removed these from young people’s lives and undermined these protective strategies.

When thinking about self-regulation, in this article I draw inspiration from Moore et al. (2017) work on drug user assemblages, which considers drug use as a product of various interconnected factors, including social, environmental, and individual influences. Their work provides strategies that their participants employ for maintaining health and well-being alongside their drug use, such as planned social activities or demarcations such as going to work. These strategies were reflected with participants of this study who were able to access these and other protective mechanisms during the pandemic, reinforcing that often, drug use and health can be found within the same assemblage. Other participants, however, did not experience the same amount of separation of these two realms due to conditions of lockdown. As a result, they faced challenges managing or supporting their physical and mental health and drug use.

The disruption of routines and lack of peer support played noticeable roles for those who reported using drugs in more uncontrolled ways. In this article, I explore the ways lockdown created fertile grounds for developing uncontrolled drug use of cannabis, alcohol, and cocaine, drugs that are already intertwined with their everyday lives. By contrast, those who were able to manage their drug use by creating new protective mechanisms or having social contact with close relations reported being able to continue using drugs during lockdown in a controlled manner.

## Methods

I draw on longitudinal qualitative interviews that were held with young people who use drugs, initially from February 2018 to April 2019 at King’s College London, London, UK, which was the host institution of this study. Thirty-one participants were recruited from the database of the London cohort of ImagenPathways,^2^ a multi-sited mixed methods study that explored pathways to uncontrolled drug use via brain imaging, hair samples, and quantitative surveys. The participants were recruited based on their responses to the Imagen timeline follow-back instrument, which tracked their alcohol and other drug consumption over the past 60 days. If they reported more than three illicit drug use events within that time, they were selected to participate. I followed their drug use over a period of 18 months via thematic semi-structured interviews that explored the kinds of illicit drugs used, the motivations for use, temporal patterns, sources of information on safer drug use, and negative and positive drug use experiences.

The second phase, and the resulting data that contributed to this article, took place from October to December 2020 and consisted of follow-up interviews with 14 of the original participants, three women and 11 men.^3^ They were approached via a messaging service used in the previous study phase. I conducted online in-depth, semi-structured interviews which focused on the pandemic’s impact on their drug using lives. During the first part of these interviews, participants were asked to provide an update on their personal life and their drug use leading up to March 2020, in order to grasp their situation and practices before lockdown. Prior to the pandemic, participants were using a variety of drugs, including cannabis, cocaine, ecstasy, alcohol, and ketamine in various settings: at home alone, at house parties with friends, at clubs and pubs with strangers, at work with colleagues, while on holiday. Follow-up interviews revealed that these patterns of the drug use had shifted, particularly in regards to alcohol, cannabis, and cocaine use.

The non-drug related updates the participants shared varied considerably: they had experienced breakups, started new relationships, moved in with partners, bought houses. They had lost elderly family members to Covid-19, and had seen parents go through cancer treatment. Some were furloughed, others were promoted. One participant lost their job and had to move back in with their parents. Another was diagnosed with burnout and with full pay, was put on doctor-appointed stress leave until June 2021. All but two participants were working from home at the time of the interviews. Further detail on participants’ information, including a comparison of the drugs used before and during the pandemic which indicate that habitual drug use increased while recreational drug use decreased, are provided in Appendix 1.

For some, 45 minutes was enough time to share how they were experiencing the pandemic; however, participants who appeared to be struggling more with the conditions, such as isolation or anxiety, talked for closer to 90 minutes. A photo elicitation approach was also used and proved to be almost as popular as during the first phase of interviews. While young people were no longer in typical settings such clubs, parties, and vacations where they are primed to use their cameras and social media apps, they still relied on their smartphones while in lockdown, and the solitude of their substance use, mostly cannabis and alcohol, was reflected in the photos that they shared.

Methodological challenges for this second phase were mainly virus-related restrictions which we navigated by conducting the follow-up interviews via secure end-to-end encrypted online platforms. While virtual interviews were not ideal from the perspective of nurturing a deep ethnography, the repeated interviews in the initial phase helped to nurture long-term relationships and created good rapport with participants. Additionally, participants were enthusiastic for the connection and platform to enable them to share their experiences while in the middle of a lockdown. Interviews were transcribed in full, coded thematically, and analyzed using NVivo software. Below, pseudonyms are used and all identifying or sensitive information was removed.

## Changes in drug use patterns

There is evidence to suggest that individuals who were stripped of the protective mechanisms of routine and peer networks developed practices of uncontrolled drug use, developed dependency, and experienced poor mental health. Influenced by this, I examine the practices of three commonly used drugs – cannabis, alcohol, and cocaine – and the patterns that developed around their use during the first year of the Covid-19 pandemic. I also include a narrative that provides a cautionary tale as to what may happen when young people lose complete access to their protective mechanisms. Firstly though, I explore how protective mechanisms worked during lockdown, and what elements participants were able to access or create in order to deal with the impact of lockdown on their drug use. By doing so, we can see more clearly the role of protective mechanisms in distinguishing uncontrolled drug use from controlled use.

### Increase in daily habits, decrease in recreational habits

Prior to the pandemic, participants reported using popular ‘party drugs’ such as cocaine, ketamine, LSD, MDMA/ecstasy, cannabis, and mushrooms when ‘going out’. There was a sharp decrease in the use of many of these drugs after March 2020 as a direct outcome of Covid-19 restrictions, such as lockdown, as nightlife became non-existent.

Joseph (24) stopped using MDMA with the arrival of Covid-19 for several reasons. First, the opportunity to do so, in the spaces he associated with its use, was no longer an option due to the conditions of the pandemic. Second, the stimulating and socializing effects of the drug were not suited to the constrained physical, social and emotional contexts of lockdown:

MDMA, that is more like a party going out kind of thing, but the lifestyle I'm living now has changed. So as a result, what is the most appropriate drug, what would be the thing that makes the most sense for me to do, I'm sitting at home, I'm chilling out. So, either smoking weed or maybe drinking some alcohol would be fine.

Joseph reflected at the start of the pandemic, he “went a bit nuts” in terms of smoking cannabis: “I'd wake up and I would smoke weed all day long.” However, by the summer of 2020, he realized that “you know, this is crazy, like, I can’t be just smoking weed all day, for however long this is gonna go, I was spending so much money, it was ridiculous.” In addition, however, he built a new daily routine with his mother that focused on a healthy mind and body, and this influenced his use of cannabis:

I toned it back a bit, and I decided ‘let’s get you get a bit healthier’. So, try and instill some good, positive things. And I bonded with [my mum], we’ll meditate together, we’ll do a little yoga, or we’ll do some exercise together in the morning. And that was kind of like our time and we’d motivate each other. So it kind of became, in essence, the new kind of ‘thing’ that we were doing for a couple of months, which was great.

Staying motivated helped him to focus on making healthy choices, but what appeared to be most useful here, something not available to all participants, was living with someone who cared about them, able to witness their decision to decrease their drug use, and encourage them into new routines.

Jesse (27), who reported frequent drug use with his peer group during the main study, reported a significant decrease in use of all drugs during the pandemic. He lives with his parents and much like Joseph, this built-in support system helped to create and maintain a healthy routine of daily exercise and leisure activities.

I get up at like, half, six in the morning, I do yoga with my mom for half an hour. I was actually doing it [yoga and biking] every day, you, getting into a routine. I like being in routine.…I've listened to podcasts most of the time. And then I've been cooking more. Me and my dad used to get in a lot later than my mom did, so she’d always cook. But we’ve been taking it in turns to cook now…lockdown has done me some favors… my relationships got better with my parents.

Developing new hobbies and spending more time with his family encouraged him to reach a place where he has more control over his drug use: “You see, I used to do it a bit too much and spend too much money. But I’ve think I’ve grown up. Slowed down. So more like a treat, if anything, rather than a habit.”

Participants reported the largest increase in use of cannabis and alcohol during the first year of the pandemic. These drugs were not necessarily tied to one particular setting or one particular activity, and their use could more easily become habitual and repetitive. Participants were able to enjoy cannabis, alcohol, and to a lesser extent, cocaine, as much at home alone as they did in pubs, clubs, or other spaces in which they socialized. Throughout the first year of the pandemic, participants reported using these drugs alone, with roommates and romantic partners, or when regulations were eased, with a small group of friends during or after visiting the pub. For individuals who smoked cannabis regularly, this often solitary practice appeared to be well-suited to home use, and for some, while they were working from home. Those who drank alcohol tended to drink as a coping mechanism for the uncertainty and stress related to the pandemic, but also as a suitable and acceptable addition to increased socializing online, such as virtual happy hours. For those who used cocaine, practices shifted during the pandemic as it was reported being used more indiscriminately. In the following, I explore the ways these non-niche drugs were being consumed in detail.

### Cannabis: protective mechanisms at work

Adrian (25), an amateur athlete working from home, used more alcohol and cannabis during the stay-at-home orders. He started smoking cannabis in the afternoons during workdays:

It went up drastically at the beginning. Like a lot. It turned into sort of like, every day…Just because I could. I had no excuse to not be smoking. I had no exams. I was at home. So, like, I would work from home every day, I would have like a desk, my desk that I'm working from, and then I'd spark up a joint.

Adrian was happy that returning to the office assisted him to (re)develop his relationship with cannabis. While he was able to smoke during his lunch break at home with little repercussion regarding his work performance, being back at the office with colleagues changed that:

Just because I won’t smoke before I go to work. I won’t, I can’t, not in the morning. And then like I wouldn’t take any to work. So that’d be a return to smoking in the evenings only Monday through Friday … obviously I wouldn’t be getting high and going in.

Adrian’s work environment, rather than an internal motivation, appeared to control his behavior. Getting high before or during working hours, a habit he developed during lockdown, became unacceptable when he returned to the office, leading to a change in his behavior. This highlights the importance of external factors, like routines and peer networks, in helping individuals control their substance use.

### Cannabis: lack of protective mechanisms at work

Before the pandemic, Klaas was a daily cannabis user, typically smoking several joints in the evening. He did not like being stoned in the London tube while commuting to work, however, and he decided to not smoke in the morning before work but to save the experience for the end of the day. Furthermore, Klaas did not get high during his working hours pre-Covid. He considered it “crazy” to do so in front of his colleagues. If he knew he had something important that day, such as a family dinner, he would not use cannabis beforehand, and weekly dinners with his girlfriend’s family also controlled his regular use. Klaas’ drug using narrative suggested signs of habitual but controlled drug use: he implemented aspects of his daily routine to delineate when and where he would and would not smoke cannabis.

Like almost all participants, Klaas was required to work from home from March 2020. Overnight, he found himself no longer having to commute, not having colleagues around, and no family dinners. In other words, he found himself without all the protective mechanisms he had come to rely on to control his cannabis use. How did the removal of these structures play out in his practices?

Klaas reported a significant increase in cannabis use in our follow-up interview. As a direct result of the looming lockdown, he ‘stocked up’ on cannabis and bought three ounces instead his regular amount of one ounce. While this supply was intended to ensure he had enough in case he couldn’t access his dealer, he ended up “smoking this big bag of weed” much sooner than intended.

During the pandemic, he smoked first thing in the morning, before work. Without having to factor in commuting time, he was afforded more time for a recreational and relaxed morning: “I would just be sitting here, bonging, watching some TV and then I’d be like ‘Oh shit it’s 9:30 I have a meeting!’ And then I’d sit down and try to be like, normal but I’m probably slower and shit than I should be.” Once he had smoked his first joint, he became more prone to continue smoking during the day – “And I'm happy to in the afternoon if things are slowing down” – and into the evening.

Dave (24), a daily cannabis user, similarly spoke of the strong association between smoking cannabis and working from home: “Just because, obviously I'm working from home all day…I don’t mind having like a smoke at lunch. And I'm happy to like in the afternoon if things are slowing down, and I've just got a task on my own. Yeah, like I can still get my work done. Still, like, it’s not like I don’t see it as an issue.”

Klaas’s and Dave’s practices reflect how the environment of working from home was conducive to daily cannabis use. Klaas had more free time in the mornings, which he used to watch TV and sometimes mindlessly smoke; he didn’t need to stay sober to deal with London morning rush hour. In addition, for both men, the solitariness of their work (coding) required little human interaction. Their companies did not typically use cameras during meetings, so neither needed to look or act ‘normal’ when talking to colleagues. As a result, they could easily spend their entire workday, unsupervised and stoned.

Klaas no longer had colleagues around to curb his habit or demonstrate the acceptability (or unacceptability) of his drug use, but also, he spent almost all his time, including for work, in the same space (his apartment) where he normally gets high. Being stuck at home seemed to encourage increased use: “I find it hard to not smoke cannabis. Because it’s there…it’s habitual, it’s a comfort.” While the setting proved to be mentally challenging, Klaas describes the continual use of cannabis extremely tempting. It was a source of “comfort” during an unsettling time. His reported increase in use created more instances such as the following to occur, situations that he referred to as a “nightmare:”

When it’s [cannabis] there and I want it, I don’t stop myself. Even if I have the thought, halfway, I’m like ‘Oh you’ve committed now,’ that’s what I tell myself, like, ‘You’re fucking rolling it now, so you might as well finish rolling. And you’ve finished rolling it so you might as well smoke it… you’re not going to leave it there, are you?’ Sometimes I will spark it, get two pulls in and I can feel myself getting high and I’m like, ‘Why are you sitting here and smoking this here? Wait 6 hours and you can have a bigger one,’ but I’ll still sit there and I’ll even have that same thought process and smoke the rest of it instead of just putting it out, having it later or just throwing it away.

Through his description of a typical day, we see how lockdowns and working from home both removed his protective mechanisms and contributed to an increase in smoking. His internal negative dialogue and constant re-evaluation was not only exhausting but suggested an unhealthy relationship. He struggled to control this, which was particularly visible when probed about working under the influence:

Like, I don’t want to get high before or during work, there is no need. I’m better at work when I’m not high. I mean I can function; I can do my job, I can write code, but it takes me longer to understand, yeah, just a bit more dopey and there’s no need to be like that at work.

While Klaas, and to some extent Dave, could manage working while high, they preferred to avoid it because it led to underperformance. This behavior mirrors the pattern seen in individuals who recognize that certain actions negatively impact their lives yet continue to engage in them, highlighting the complexities and challenges of controlled use. Relatedly, it could be suggested that the constant negotiation Klaas faces with his daily habits—such as avoiding smoking during work to prevent feeling 'dopey’ and recognizing 'there’s no need’ yet ultimately failing to control the urge—may have contributed negatively to his mental well-being.

In comparison to Adrian, who was able to rely on external protective mechanisms such as going to work and being around colleagues, Klaas had to use an internal force, his own willpower, to try to cut back on smoking cannabis during the workday. To help enforce this desired change, he enlisted his girlfriend’s help:

I’ve asked my missus to take my weed, hide it in the flat; whatever you do, don’t tell me where it is, ‘cos soon as I know where it is, I’ll just go get it. It’s weird, once I have the thought, there’s no stopping me. If it’s there, I won’t stop thinking about it, it drives me nuts.

This struggle exemplifies the broader challenges individuals face when trying to self-regulate substance use without external support systems.

### Alcohol and cocaine: shifting practices

All participants who reported drinking regularly before the pandemic reported spikes in their alcohol use in our follow-up interviews. Reasons ranged from boredom of lockdown (“When it first started, we were probably drinking more, because we were just at home like, watching Telly,” Nicole, 24) to excitement in-between lockdowns when bars were open again (“all the pubs in England sort of reopened and the restrictions were mostly lifted. So, it’s obviously quite a lot of catching up to do,” Tim, 25).

Alcohol was seen and chosen as the desired (and accepted) substance to use alone or with partners, or to accompany the spike in ‘virtual happy hours’ with colleagues, (“we did have a few Zoom calls of mates and things like that, a few got quite boozy,” Mortimer, 24) or long-distance check-ins with family and friends: “Me and my partner did find that one of the ways to sort of you know, sort of spend time when you were locked in was to have a few more drinks, and weekdays and we’d have like Zoom calls with family and we’d socialize with drinks” (Jeff, 25).

Like most other participants, Kiera (25) noted an increase in alcohol use, particularly during the first lockdown from mid-March to mid-May: “In regard to lockdown, I went through a stage of drinking an awful lot. Yeah, but it really, really increased. I was like, I should probably chuck the drinking because I was drinking like two bottles of Prosecco a night… Yeah, I would say it’s probably increased quite dramatically.”

As explored elsewhere (Murray 2023) with this same group of young adults, the presence of alcohol often encouraged the use of another perceived low risk, low threshold drug – cocaine. Instead of coming home after a busy week away, Kiera now conducted all of her work from home with little demarcation between when work ended and recreational time began. Kiera found herself using cocaine every now and then to make her monotonous life under lockdown feel a bit more “special”. Kiera also found that the conditions of lockdown and being stuck at home encouraged new ways of using cocaine.

Like Klaas and Dave, the backdrop of being stuck at home must also be considered to better understand the impact of the pandemic on Kiera’s drug use. Before the pandemic, there was a clear separation between work and leisure, and this may have helped prevent such behaviors. She described a scenario that working from home created: the blurring of working days and weekends, ‘work time’ and ‘play time.’ She shared that without anyone to look over her and hold her responsible, or tell her what is acceptable behavior on weeknights versus weekends, she could very easily find her drug use slipping out of control: “Like, ‘Friday nights’, for example, that’s not really a thing. So, it’s like, during lockdown, I'm gonna still take coke on Friday. At a certain point, it’s like, why don’t I just take coke on a like Tuesday morning? Like, who cares?” “*Who cares*?” is an important comment for someone who spent the first lockdown alone.

It is also useful to consider some characteristics of cocaine, which has a high potential for dependency: it is ubiquitous in young peoples’ social lives; it has perceived low harm; and it is frequently used in connection with alcohol, as these two drugs are seen as compatible (Murray 2023), and has high acceptability of use in a multitude of settings. Although alcohol is licit and cocaine illicit, both are easily accessible, perceived low-threshold, low-risk drugs. In addition, cocaine is seen as less disruptive to everyday routines. It can be integrated into an individual’s daily life quiet easily, as Kiera demonstrated when she suggested that she saw the possibility to use it on a Tuesday morning before work.

These narratives provide a compelling argument that being stuck at home encouraged behaviors that would not necessarily be exhibited if they still had the built-in protective mechanisms, such as going to work or the gym, or having someone close by to check in.

By January 2022, Kiera, Dave, and Klaas, like many others, were in a similar situation to that when we spoke in October 2020: a return to the colder months, partial lockdowns, and working from home. This setting encouraged prolonged and regular use without peer support.

### Opioids: a cautionary tale

During the first phase of the study, MacMillan (25) relied heavily on prescribed opioids (such as benzodiazepines) during the week and saved using illegally bought heroin to use at home on weekends. His protective mechanism was that he would only use heroin on weekends when he had longer stretches of time to use and more time to recover. During our follow-up interview, MacMillan reported that he had been put on full paid sick leave due to burn-out in April 2020 and was living alone. He had moved out of the apartment that he had shared with a friend due to a disagreement, his romantic relationship had ended, and he was now spending all of his time alone, without work but with an income, with very little connection to the outside world, and in constant pain. He was looking up ways to shift from smoking heroin to injecting it, a route of administration that brings more threats to an individuals’ health: “I do have all the equipment ready. I don’t really know what’s stopping me to be honest it’s kind of like the same thing that stops me from doing everything else, it’s just like lack of motivation.”

While MacMillan claimed that his chronic depression instigated his daily opioid use, a practice over many years, it also contributed to his lack of motivation to switch from smoking to injecting the drug. Additionally, his depression exacerbated the isolation and challenges he faced during lockdown, leading to a cycle of worsening mental health. However, the most crucial element was losing his job and no longer having a daily routine.

It was the being cut off from everyone, being at home, because I think my life was really just like, shambolic, just a mess that was, it was like a Jenga tower [tower of wooden blocks] you know? One piece and it just comes crashing down and the only thing that was holding it up was, I was forced into a routine of getting up in the morning, brushing my teeth, having a shower, going to work. You know, that was what was basically holding my life together. And then without that routine forced on me, you know, the supports on the Jenga tower just came apart and it just crashed down. I think I would still be at work but I would still be depressed. But I would be dealing with it, because I would have to, I’d be forced to deal with it.”

MacMillan describes how essential a daily routine was for him to keep moving forward, face responsibilities, and take care of his personal hygiene and well-being. The seriousness of his situation and the removal of the ‘supports of the Jenga pieces’ was very real; a few times he expressed suicidal ideation. His narrative serves the argument that without external or internal factors, lockdown created extraordinarily dangerous circumstances for some, particularly for young adults struggling with their mental health.

## Conclusion

Building upon previous research into drug use trajectories, I examine how times of uncertainty impact the ability of people who use drugs to control their use. During the pandemic, participants faced new challenges in controlling their drug use, such as trying not to get high while working from home. One contributing factor to this shift was that the drugs reported as harder to control have characteristics making them more suitable and likely to be used regularly during lockdown. The perception of alcohol, cannabis, and to a lesser extent cocaine are that they are lower-risk drugs, a view influenced by their relatively normalized, prevalent use and in the case of alcohol, its legal status. Another factor was the increased isolation participants experienced, impacting their behavior and patterns around drug use. Harm reduction from below, which relies heavily on social connection and self-regulation techniques for maximizing pleasure, was disrupted during the pandemic and participants who were isolated were more vulnerable to encounter challenges related to their drug use. Their experiences with cannabis, alcohol, and cocaine show that without the protective mechanisms of daily routines—like responsibilities and social connections—some individuals reported losing control.

To help address these changes, suggestions would be to increase support in finding more sustainable and manageable routines, as well as ensuring access to mental health support services that address not only uncontrolled drug use but the impact of social isolation and loneliness as well. These efforts underscore the need to raise awareness of the potential negative effects of isolation, particularly for young people who use drugs.

There were participants who were able to create and rely on new ways to control their drug use during the pandemic. They were able to create sustainable routines that prioritized health, social connection, and accountability. This reminds us of how crucial peer groups and intimate relations are for encouraging safer, more enjoyable drug use. It also reinforces the strength of a ‘harm reduction from below’ framework when examining young people’s drug using lives.

For those who do face obstacles with their drug use influenced by instability and isolation, we – as drug researchers looking to support those we study – need to understand if and what behaviors they are looking to change and how those changes can be supported. It is essential that we understand and continue to explore the ways in which drug use practices are evolving and how times of uncertainty and isolation impact young people in terms of drug use practices and overall well-being.

## Notes

1 The UK’s first lockdown occurred from late March to June 2020, the second lockdown began in early November 2020, and the third lockdown lasted from January to early March 2021.

2 Funded by the European Research Area Network on Illicit Drugs (ERANID), ImagenPathways aimed to understand the interplay between cultural, biological, and subjective factors in drug use pathways. This project built upon the extensive data collected by the IMAGEN project, which followed over 2,000 Europeans from adolescence.

3 Eight participants choose to withdraw before completing the three interviews in the first phase, so of the 21 that completed the first phase, seven did not reply to my request for a follow-up interview during the pandemic.

## Appendix 1: Overview of participants situation during time of interview

**Table:**

Participant pseudonym	Age	Occupation	Living situation	Drugs used before pandemic (in order of 1^st^ use), bold indicates regular use (more than 2x in lifetime)	Drugs use during pandemic/time of interview
Adrian	24	Working part time, Studying accounting	Recently single and lives with housemate, moved to Wales	Alcohol, cannabis, ecstasy, cocaine, ketamine	Alcohol, Cannabis (daily), cocaine
Andrew	24	Furloughed (from airport land surveying)	Single, lives with housemate, moved to Edinburgh	Alcohol, Cannabis, mushrooms, ecstasy	Alcohol, Cannabis
Astrid	24	accountant	Lives with boyfriend, buying a house in London	Alcohol, cannabis	Alcohol, Cannabis (stayed the same)
Dave	24	Software engineer	Lives with girlfriend in East London	Alcohol, cannabis, ecstasy, ketamine, cocaine, methamphetamine, mushrooms, LDS, 2CB, salvia, benzodiazepine, NPS′* (new psychoactive substances)	Alcohol, Cannabis, cocaine, ecstasy, ketamine, mushrooms
Jeff	25	Masters in Data Science, working in finance	Lives with girlfriend in flat in South London	Alcohol, cannabis, cocaine, ecstasy, ketamine, LSD, mushrooms	Alcohol, cocaine
Jesse	27	Structural engineer	Recently single, in long distance relationship, lives with his parents in Southeast London	Alcohol, cannabis, cocaine, ketamine, ecstasy, benzodiazepine	Alcohol, cocaine, cannabis
Joseph	24	Dentist (furloughed first 3 months of lockdown and recording artist)	Recently single, lives at home with family in Southwest London	Alcohol, cannabis, LSD, ketamine, ecstasy, cocaine, benzodiazepine, mushrooms, 2CB, NPS	Alcohol, cannabis, LSD
Kiera	23	External sales representative for data construction	In a new relationship, lives alone in London	Alcohol, cannabis, ecstasy, ketamine, cocaine,	Alcohol, cocaine, cannabis
Klaas	25	Software developer	Lives with girlfriend in greater London	Alcohol, cannabis, ecstasy, ketamine, cocaine, benzodiazepine, methamphetamine, LSD, mushrooms, salvia, 2CB, NPS	Alcohol, cannabis, cocaine
MacMillian	24	Sickleave from work as paralegal	Recently single, bought own house in York	Alcohol, cannabis, ketamine, cocaine, LSD, ecstasy, benzodiazepines, methamphetamine, prescription opioids, heroin (rectally), NPS, GHB, crystal meth	Alcohol, cannabis, heroin (smoking), benzodiazepines, prescription opioids
Mortimer	24	Mechanical Engineering degree at University of Nottingham, not project manager for company that installs conveyer systems	In a relationship, start of lockdown moved in with family in London	Alcohol, cannabis, cocaine, ketamine, ecstasy, LSD	Alcohol, cannabis
Nicole	24	Teacher (ages 11-18)	Lives with boyfriend in Sheffield	Alcohol, cannabis, ecstasy, cocaine, ketamine	Alcohol
Shane	24	Temporary furlough relay	Lives with girlfriend in flat in Wimbledon	Cannabis, mephradone, ecstasy, cocaine	Alcohol
Tim	25	Furloughed, then laid off, hired as security guard but not yet working	Moved out of flat and in with parents in South London	Alcohol, cannabis, ecstasy, ketamine, cocaine, LSD	Alcohol, cannabis

* New psychoactive substances

## References

[CIT0001] Banken, J. A. 2004. “Drug Abuse Trends Among Youth in the United States.” *Annals of the New York Academy of Sciences* 1025 (1): 465–471. 10.1196/annals.1316.057. 15542750

[CIT0002] Beard, S. J., and J. M. Wolff. 2022. “The Moderating Role of Positive Peers in Reducing Substance Use in College Students.” *Journal of American College Health: J of ACH* 70 (4): 1059–1070. 10.1080/07448481.2020.1784907. 32669053

[CIT0003] Bengtsson, T. T., and S. Ravn. 2021. *Youth, Risk, and Routine: A New Perspective on Risk-Taking in Young Lives*. London: Routledge UK.

[CIT0004] Benschop, A., F. van Bakkum, and J. Noijen. 2021. “Changing Patterns of Substance Use During the Coronavirus Pandemic: Self-Reported Use of Tobacco, Alcohol, Cannabis, and Other Drugs.” *Frontiers in Psychiatry* 12: 633551. 10.3389/fpsyt.2021.633551. 34122170 PMC8187560

[CIT0005] Brenan, M. 2020. "Americans Say COVID-19 Hurting Mental Health Most." *Gallup*. https://news.gallup.com/poll/308420/americans-say-covid-hurting-mental-health.aspx

[CIT0006] Coggans, N., and S. McKellar. 2009. “Drug Use Amongst Peers: Peer Pressure or Peer Preference?” *Drugs: Education, Prevention and Policy* 1 (1): 15–26. 10.3109/09687639409028532.

[CIT0007] Copeland, Molly, Jacob C. Fisher, James Moody, and Mark E. Feinberg. 2018. “Different Kinds of Lonely: Dimensions of Isolation and Substance Use in Adolescence.” *Journal of Youth and Adolescence* 47 (8): 1755–1770. 10.1007/s10964-018-0860-3. 29774451 PMC6045973

[CIT0008] Decorte, T. 2001. “Drug Users’ Perceptions of ‘Controlled’ and ‘Uncontrolled’ Use.” *International Journal of Drug Policy* 12 (4): 297–320. 10.1016/S0955-3959(01)00095-0.

[CIT0009] Dewa, L. H., C. Crandell, E. Choong, J. Jaques, A. Bottle, C. Kilkenny, A. Lawrence-Jones, M. Di Simplicio, D. Nicholls, and P. Aylin. 2021. “CCopeY: A Mixed-Methods Coproduced Study on the Mental Health Status and Coping Strategies of Young People During COVID-19 UK Lockdown.” *The Journal of Adolescent Health: official Publication of the Society for Adolescent Medicine* 68 (4): 666–675. 10.1016/j.jadohealth.2021.01.009. 33589305 PMC9188746

[CIT0010] Duff, C. 2012. “Party Drugs and Party People: Examining the 'Normalization’ of Recreational Drug Use in Melbourne, Australia.” *International Journal of Drug Policy* 23 (5): 394–400.

[CIT0011] Dumas, T. M., W. Ellis, and D. M. Litt. 2020. “What Does Adolescent Substance Use Look Like During the COVID-19 Pandemic? Examining Changes in Frequency, Social Contexts, and Pandemic-Related Predictors.” *The Journal of Adolescent Health: official Publication of the Society for Adolescent Medicine* 67 (3): 354–361. 10.1016/j.jadohealth.2020.06.018. 32693983 PMC7368647

[CIT0012] Guest, E., C. Coope, and S. Oram. 2021. “The Impact of the COVID-19 Pandemic on Young People’s Substance Use in the United Kingdom: A Mixed-Methods Study.” *International Journal of Environmental Research and Public Health* 18 (4): 1962.33670481

[CIT0013] Harbers, M., T. Hulshof, and R. Schaink. 2020. “Inventarisatie Nederlandse COVID-19 Onderzoeken: Preventie en Zorg & Brede Maatschappelijke Vraagstukken.” Rapportage nr 7 Colofon.

[CIT0014] Hardon, A. 2021. *Chemical Youth: Navigating Uncertainty in Search of the Good Life*. London: Palgrave Macmillan.

[CIT0015] Hardon, A., and T. D. Hymans. 2016. “Guest Editors’ Introduction: Harm Reduction From Below.” *Contemporary Drug Problems* 43 (3): 191–198. 10.1177/0091450916663247. 27721524 PMC5046162

[CIT0016] Hardon, Anita, Takeo David Hymans, Inge Van Schipstal, Swasti Mishra, Moritz Berning, Hayley Murray, Daan Kamps, and Tait Mandler. 2020. “Caring for “hassle‐free Highs” in Amsterdam.” *Anthropology and Humanism* 45 (2): 212–222. 10.1111/anhu.12298.

[CIT0017] Hawke, L. D., S. Barbic, A. Voineskos, P. Szatmari, K. Cleverly, E. Hayes and J. Relihan 2020. “Impacts of COVID-19 on Youth Mental Health, Substance Use, and Well-Being: A Rapid Survey of Clinical and Community Samples.” *Canadian Journal of Psychiatry* 65 (10): 701–709. 10.1177/0706743720940562. 32662303 PMC7502874

[CIT0018] Hunt, G., and K. Evans. 2008. “Dancing on the Edge: ‘Normal Party Drug’ Use, Normalisation and Young People’s Narratives.” *Health, Risk & Society* 10 (3): 281–303.

[CIT0019] Kokkevi, A., C. Richardson, S. Florescu, M. Kuzman, and E. Stergar. 2007. “Psychosocial Correlates of Substance Use in Adolescence: A Cross-National Study in Six European Countries.” *Drug and Alcohol Dependence* 86 (1): 67–74. 10.1016/j.drugalcdep.2006.05.018. 16837140

[CIT0020] Layman, H. M., I. E. Thorisdottir, T. Halldorsdottir, I. D. Sigfusdottir, J. P. Allegrante, and A. L. Kristjansson. 2022. “Substance Use Among Youth During the COVID-19 Pandemic: A Systematic Review.” *Current Psychiatry Reports* 24 (6): 307–324. 10.1007/s11920-022-01338-z. 35476186 PMC9043089

[CIT0021] Mandler, T. 2016. “Producing Pleasure, Minimizing Harm: Chemical Use and Harm Reduction by Queer Nightlife Workers in Brooklyn, NY.” *Contemporary Drug Problems* 43 (3): 258–276. 10.1177/0091450916651.

[CIT0022] Mayock, P., M. L. Corr, and E. O’Sullivan. 2017. “Understanding Youth Social Behavior and Drug Use in Context: A Qualitative Study of Young People’s Drug Use in Recreational Settings.” *Drugs: Education, Prevention and Policy* 24 (2): 127–134.

[CIT0023] Moore, D., K. Pienaar, E. Dilkes-Frayne, and S. Fraser. 2017. “Challenging the Addiction/Health Binary with Assemblage Thinking: An Analysis of Consumer Accounts.” *The International Journal on Drug Policy* 44: 155–163. 10.1016/j.drugpo.2017.01.013. 28431794

[CIT0024] Murray, H. 2023. “Young Adults Pathways of Negating Harms and Pleasure Related to Recreational Poly-Drug Use: A Complex Balancing Act.” *Journal of Addiction Research* 7 (1): 9–19 10.33140/JAR.07.01.03.

[CIT0025] Nawi, A. M., R. Ismail, F. Ibrahim, M. R. Hassan, M. R. A. Manaf, N. Amit, N. Ibrahim, et al. 2021. “Risk and Protective Factors of Drug Abuse Among Adolescents: A Systematic Review.” *BMC Public Health* 21 (1): 2088. 10.1186/s12889-021-11906-2. 34774013 PMC8590764

[CIT0026] Nichter, M., and N. Taylor. 2021. *A Filtered Life: Social Media on a College Campus*. New York: Routledge.

[CIT0027] Rogés, J., M. Bosque-Prous, J. Colom, C. Folch, T. Barón-Garcia, H. González-Casals, E. Fernández, et al. 2021. “Consumption of Alcohol, Cannabis, and Tobacco in a Cohort of Adolescents Before and During COVID-19 Confinement.” *International Journal of Environmental Research and Public Health* 18 (15): 7849. 10.3390/ijerph18157849. 34360141 PMC8345772

[CIT0028] Salvatore, C. 2018. *Sex, Crime, Drugs, and Just Plain Stupid Behaviors: The New Face of Young Adulthood in America*. London: Palgrave Macmillan.

[CIT0029] Townsend, E. 2020. “Debate: The Impact of School Closures and Lockdown on Mental Health in Young People.” *Child and Adolescent Mental Health* 25 (4): 265–266. 10.1111/camh.12428. 33049100 PMC7675670

[CIT0030] Trimbos Instituut. 2020. “De Impact Van COVID-19 en de Coronamaatregelen op Alcohol-, Tabak- en Drugsgebruik Onder Uitgaanders.”

[CIT0031] Turner, N., V. Cooper, K. Hughes, S. Lenton, and J. Johnston. 2021. “The Impact of COVID-19 on Young People Who Use Drugs: Findings from a Mixed Methods Study.” *Journal of Substance Use* 26 (1): 35–41.

[CIT0032] Twenge, J. M., and T. E. Joiner. 2020. “Mental Distress Among U.S. Adults During the COVID‐19 Pandemic.” *Journal of Clinical Psychology* 76 (12): 2170–2182. 10.1002/jclp.23064. 33037608 PMC7675251

[CIT0033] Van Schipstal, I., S. Mishra, M. Berning, and H. Murray. 2016. “Harm Reduction From Below: On Sharing and Caring in Drug Use.” *Contemporary Drug Problems* 43 (3): 199–215. 10.1177/0091450916663248. 27721525 PMC5046163

[CIT0034] Woo, J., A. Bhalerao, and R. Bhatia. 2020. “Impact of the COVID-19 Pandemic on the Lifestyle and Mental Health of Children and Adolescents.” *Cureus* 12 (8): e10123.32879836

[CIT0035] YoungMinds. 2021. Coronavirus: Impact on Young People with Mental Health Needs. YoungMinds. https://www.youngminds.org.uk/about-us/reports-and-impact/coronavirus-impact-on-young-people-with-mental-health-needs/.

[CIT0036] Zinberg, N. E. 1986. *Drug Set and Setting: The Basis for Controlled Intoxicant Use*. New Haven: Yale University Press.

